# The Time-Course of Acute Changes in Corticospinal Excitability, Intra-Cortical Inhibition and Facilitation Following a Single-Session Heavy Strength Training of the Biceps Brachii

**DOI:** 10.3389/fnhum.2016.00607

**Published:** 2016-12-01

**Authors:** Christopher Latella, Ashlee M. Hendy, Alan J. Pearce, Dan VanderWesthuizen, Wei-Peng Teo

**Affiliations:** ^1^School of Exercise and Nutrition Sciences, Deakin UniversityBurwood, VIC, Australia; ^2^Melbourne School of Health Sciences, The University of MelbourneMelbourne, VIC, Australia; ^3^Clinical Exercise Science and Rehabilitation, Institute of Sport, Exercise and Active Living (ISEAL), Victoria UniversityFootscray, VIC, Australia; ^4^Institute for Physical Activity and Nutrition (IPAN), School of Exercise and Nutrition Sciences, Deakin UniversityBurwood, VIC, Australia

**Keywords:** transcranial magnetic stimulation, corticospinal excitability, intra-cortical inhibition, intra-cortical facilitation, super-compensation, strength training

## Abstract

**Objective**: The current understanding of acute neurophysiological responses to resistance training remains unclear. Therefore, we aimed to compare the time-course of acute corticospinal responses following a single-session heavy strength training (HST) of the biceps brachii (BB) muscle and provide quantifiable evidence based on the super-compensation model in an applied setting.

**Methods**: Fourteen participants completed a counter-balanced, cross-over study that consisted of a single HST session (5 sets × 3 repetition maximum [RM]) of the BB and a control session (CON). Single- and paired-pulse transcranial magnetic stimulation (TMS) was used to measure changes in motor-evoked potential (MEP) amplitude, intra-cortical facilitation (ICF), short-interval intra-cortical inhibition (SICI) and long-interval intra-cortical inhibition (LICI). Additionally, maximal muscle compound wave (M_MAX_) and maximal voluntary isometric contraction (MVIC) of the BB were taken. All measures were taken at baseline, immediately post and at 10, 20, 30 min and 1, 2, 6, 24, 48 and 72 h post-training.

**Results**: A significant reduction in MEP amplitude was observed immediately post training (*P* = 0.001), while MVIC (*P* < 0.001) and M_MAX_ (*P* = 0.047) were reduced for up to 30 min post-training. An increase in MVIC (*p* < 0.001) and M_MAX_ (*p* = 0.047) was observed at 6 h, while an increase in MEP amplitude (*p* = 0.014) was only observed at 48 and 72 h. No changes in SICI, ICF and LICI were observed.

**Conclusion**: Our results suggest that: (1) acute changes in corticospinal measures returned to baseline in a shorter timeframe than the current super-compensation model (24–48 h) and (2) changes in corticospinal excitability post-HST may be modulated “downstream” of the primary motor cortex (M1).

## Introduction

It is well-documented that repeated sessions of heavy strength training (HST) induces lasting adaptations at many levels of the neuromuscular system (Sale, 1988; Aagaard et al., 2002; Carroll et al., 2011) resulting in overall strength gains (for review see Zatsiorsky, 2008). In particular, adaptations to the central nervous system such as an increase in corticospinal excitability and release of short-interval intra-cortical inhibition (SICI) following 2- to 8-week strength training programs have been commonly observed (Deschenes et al., 1994; Kidgell et al., 2010; Latella et al., 2012; Weier et al., 2012; Hendy and Kidgell, 2013). While there is strong evidence to suggest that significant neural adaptations occur following multiple resistance training sessions (Kidgell et al., 2010; Latella et al., 2012), and acute changes in corticospinal excitability with sustained submaximal isometric exercise (Nuzzo et al., 2016), few studies have systematically investigated the acute central and peripheral neural responses associated with a single HST session.

Previously, acute corticospinal responses following a single-session of exercise is thought to reflect central fatigue or acute neuroplastic responses to exercise (Smith et al., 2007; Teo et al., 2012). These studies have commonly showed a reduction in corticospinal excitability, as measured by a decrease in motor-evoked potential (MEP) amplitude, and an increase in SICI following maximal and submaximal exercise. Further, peripheral changes such as a reduction in motorneurone excitability and maximal strength production have also been reported (Todd et al., 2003). While these studies provide some insights into the initial corticospinal and peripheral responses to exercise, they only provide a short “window” of observation to the neural responses, mostly only up to 60 min post-exercise, which limits our understanding of the time-course and recovery of neuromuscular system following HST.

To better understand the time-course and body’s physiological responses to exercise, Bompa and Haff (2009) previously proposed the super-compensation model that consists of four distinct phases: (1) fatigue (0–2 h); (2) compensation back to baseline (24–48 h); (3) super-compensation beyond baseline (48–72 h); and (4) involution (>72 h). They suggested that while the recovery period (i.e., fatigue and compensation phase) may differ depending on the type of exercise and intensity, more neurally-demanding exercises, one such as HST, may require up to 24–48 h to recover back to baseline and for super-compensation to occur. However, to the best of our knowledge, no studies to date have investigated the time-course of corticospinal responses following HST and compared it to the current super-compensation model.

Therefore, the purpose of this study was to map the acute time-course of corticospinal excitability, intra-cortical inhibition and facilitation, peripheral nerve excitability and maximal force production of the biceps brachii (BB) up to 72 h following HST. Specifically, we aim to determine if the changes in corticospinal and peripheral responses would coincide with the four stages of the super-compensation model proposed by Bompa and Haff (2009). Based upon evidence that strength improvements may still be observed even if HST was performed with less than 48 h rest in between HST sessions (Raastad et al., 2012; Cook et al., 2014), we hypothesized that the fatigue, compensation and super-compensation phases of the cycle would be shorter than current suggestions of a 24–48 h recovery period back to basal levels.

## Materials and Methods

### Subjects

Fourteen healthy (7M, 7F) right-handed participants (age 26.2 ± 5.8 years, height 179.2 ± 3.8 cm, body mass 79.1 ± 15.9 kg) participated in the study. Prior to transcranial magnetic stimulation (TMS) all participants were screened using a TMS safety questionnaire to exclude participants with potential contraindications, such as implants in the skull, previous history or head trauma, concussion or seizures, use of prescribed medications or the presence of any neurological disorders prior to testing (Rossi et al., 2009). To rule out a further confounding variable of age-related response to TMS, criteria of 18–35 years of age had to be met. All participants were tested at the same time-of-day and were asked to refrain from consuming caffeine 24 h prior to and during the study.

All participants were recreationally resistance-trained (at least 6 months experience) with no reported incidence of neuromuscular injury to the upper limb and reported training at least twice per week (average 3 h weekly total). A recreationally trained population was chosen to rule out possible lasting effects of excessive delayed onset muscle soreness; novice populations, or a ceiling effect; experienced populations and therefore deemed to provide a more accurate representation of a typical super-compensation paradigm. Informed written consent was obtained for each participant prior to the start of testing session. Test of limb dominance was conducted using the Edinburgh handedness test (Oldfield, 1971) and the dominant limb was used for all testing conditions. This study was carried out in accordance with the recommendations of Deakin University Human Research Ethics Committee with written informed consent from all subjects. All subjects gave written informed consent in accordance with the Declaration of Helsinki. The protocol was approved by the Deakin University Human Research Ethics Committee (Project ID: 2013-198).

### Experimental Protocol

All participants completed the study, performing HST and control (CON, no training) sessions in a randomized, counter-balanced order. Participants completed a familiarization session to introduce the single-arm dumbbell curl exercise of the BB and TMS procedures to reduce any potential effect of learning. HST was performed using a standard preacher curl bench (Life Fitness, Mulgrave, VIC, Australia) and weight adjustable dumbbell (Australian Barbell Company, Mordialloc, VIC, Australia). TMS was conducted with the participant seated in a standard desk chair. Each participant’s 1 repetition maximum (RM) was also determined during the familiarization session. A 1-week washout period was implemented after familiarization and between conditions (HST vs. CON). The contraction tempo for the BB contractions was set at 3 s eccentric phase, 0 s pause, 3 s concentric phase and has been previously used in other strength training studies investigating neurophysiological outcomes (Latella et al., 2012; Weier et al., 2012; Hendy and Kidgell, 2013). Participants were asked to refrain from exercise 72 h prior to and during the course of each condition.

**Figure 1:**
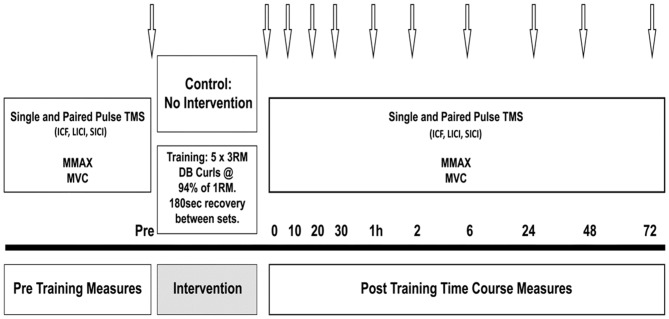
**Schematic illustration of the protocol.** Arrows indicate testing time points in minutes or hours post-training.

Prior to the training session, all participants performed a 5 min warm up on a cycle ergometer at 60% estimated maximum heart rate, and two warm up sets of 12 and 10 repetitions with increasing weight. The training load for the HST was set at the participants’ estimated 3 RM, calculated as a percentage (90–95%) of the 1 RM obtained in the familiarization session (Bompa, 1999). Working sets consisted of five sets of 3 RM with 180 s recovery between each set. The training load was increased if the researcher (a certified strength and conditioning practitioner) deemed that extra repetitions could be performed, and likewise, lowered if failure to complete the repetitions with proper form was observed. For CON, all participants performed the pre training measures, then sat quietly for 20 min, corresponding to the exercise duration in the strength condition, then performed post measures at the same time points-baseline, immediately post, 10, 20 and 30 min, and again at 1, 2, 6, 24, 48 and 72 h (see Figure 1).

### Maximal Voluntary Isometric Contraction (MVIC) of the Elbow Flexors

Maximal voluntary isometric contraction (MVIC) of the elbow flexors was measured using a hand-held force transducer (Powerlab, Inc., Terrell, TX, USA) at each time point following the HST protocol. Participants performed three slow ramp MVIC trials against an immovable resistance with the arm resting on a platform while maintaining 90° of elbow flexion, as measured by a goniometer (Biometrics, USA). Verbal encouragement and visual feedback were given for each maximal effort until no further increase in force was observed and the highest recorded force of the three trials was reported.

### TMS Measurements

All TMS measurements pre- and post-training were taken with the participant seated with their arm resting at a 90° angle. Surface electromyography (sEMG) was recorded from the BB muscle in the right arm using Ag-AgCL electrodes. Two electrodes were placed 20 mm apart on the midpoint of the belly of BB, with the ground electrode placed over the lateral epicondyle of the right radius. The skin was prepared by removing any hairs and cleaned with 70% isopro alcohol swabs prior to the placement of the electrodes. sEMG signals were amplified (1000×) with bandpass filtering between 20 Hz and 1 kHz and digitized at 10 kHz for 500 ms, recorded and analyzed using PowerLab 4/35 (ADinstruments, Bella Vista, NSW, Australia).

To ensure consistent delivery of TMS stimuli within and between testing sessions, all participants wore a snug-fitted cap (EasyCap, Germany), positioned in relation to nasion-inion and inter-aural lines and re-fitted each session in line with these measurements to ensure consistency across all time points. The cap was marked with points at 1 cm intervals in a longitude-latitude matrix, to allow repeated stimuli to be performed at the same point over the motor cortex each time. The cap was checked regularly (after every 20 stimulus) to ensure that no changes in position occurred.

Single and paired-pulse TMS was applied over the motor representation of the BB on the primary motor cortex (M1), using a 70 mm figure-of-eight coil attached via a BiStim unit (Magstim 200^2^ Magstim, Dyfed, UK). Sites near the estimated center of the BB area were explored to determine the spot at which the largest and most consistent (at least 5 out of 10 trials) MEP amplitude was evoked. This site was defined as the “optimal” site. The TMS coil was placed tangential to the skull (Latella et al., 2012) with the handle tilted 45° away from the midline while delivering TMS (Di Lazzaro et al., 2004).

All TMS measures were recorded from the BB at rest with background sEMG 100 ms before stimulation analyzed to ensure no activation. Resting motor threshold was first determined by delivering 10 TMS pulses that elicited a peak-to-peak MEP amplitude of 0.05–0.1 mV in 5 out of 10 pulses. Ten single-pulse TMS were then applied at 20% above RMT (120% RMT) with a random inter-stimulus interval of 5–8 s. All single-pulse MEP amplitude was normalized to the maximum compound wave (M_MAX_) and reported as a ratio of M_MAX_ (MEP amplitude/M_MAX_). Paired-pulse TMS consists of a conditioning (CS) and test stimulus (TS) separated by individual interstimulus intervals (ISI) used to analyze SICI, intra-cortical facilitation (ICF) and long-interval intra-cortical inhibition (LICI). The paired-pulse TMS configuration for SICI, ICF and LICI were as follows: SICI (CS = 90% RMT, TS = 120% RMT, ISI = 3 ms; Kujirai et al., 1993), ICF (CS = 90% RMT, TS = 120% RMT, ISI = 12 ms; Kujirai et al., 1993; Kobayashi and Pascual-Leone, 2003) and LICI (CS = 120% RMT, TS = 120% RMT, ISI = 100 ms; McNeil et al., 2011b; Du et al., 2014). Both SICI and ICF were expressed as a percentage of the unconditioned single-pulse MEP amplitude, while LICI was calculated and expressed as a percentage of the test to conditioning MEP amplitude for each individual paired stimuli.

### M_MAX_ Measurements

M_MAX_ was obtained from the right BB muscle by direct supramaximal electrical stimulation (pulse duration 100 ms) of the musculocutaneous nerve under resting conditions using a high-voltage constant current stimulator (Nihon Khoden, Japan). Stimulation was delivered by positioning bipolar electrodes over the right brachial plexus (Hendy et al., 2015) at Erb’s point. An increase in current strength was increased progressively until there was no further increase in sEMG amplitude. To ensure maximal responses, the current was increased an additional 20% and the average M_MAX_ obtained from five stimuli was recorded.

### Statistical Analyses

All data analyzed using IBM SPSS Statistics (IBM, Armonk, NY, USA). Data was screened with a Shapiro-Wilk test and found to be normally distributed prior to further analysis. One-way repeated measures (1 × 11) analysis of variance (ANOVA) was used to compare changes in outcome measures (MVIC, M_MAX_, MEP, SICI, ICF and LICI) across time points (Pre × Post, Pre × 10 min, Pre × 20 min, Pre × 30 min, Pre × 1 h, Pre × 2 h, Pre × 6 h, Pre × 24 h, Pre × 48 h, Pre × 72 h) for HST and CON separately. This approach, of using a one-way ANOVA was used as the main aim of the study was to map the time-course of neurophysiological changes post-HST and not a between-group comparison and is similar to other acute (Kumar et al., 2012) and physiological studies tracking responses over time (DeFreitas et al., 2011). Where statistical significance was detected, *post hoc* *t*-tests with a Bonferroni correction were conducted to test for changes to baseline measures (Field, 2013). Alpha level was set at *P* < 0.05, and all results are displayed as MEAN ± SD. Where significance was not met, but approached the alpha level (*p* ≥ 0.05 ≤ 0.07), effect size was calculated using Cohen’s *d* formula:

(1) $$\text{Cohen’​s }d\;\text{=}\;M_{\text{1}}\;\text{−}\;M_{\text{2}}{\text{/SD}}_{\text{pooled}}$$

Calculations were grouped into moderate *d* ≥ 0.5 < 0.79 or large *d* ≥ 0.80. Only interactions with a moderate or large effect sizes were reported in the analysis.

## Results

### Maximal Isometric Force Production

**Figure 2:**
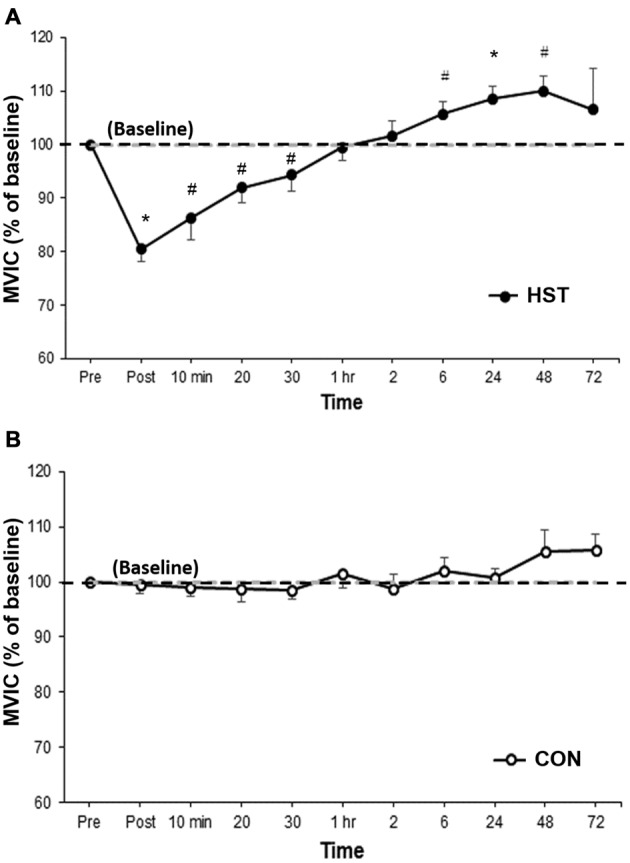
**Changes in maximal voluntary isometric contraction (MVIC) as a percentage of baseline for (A)** heavy strength training (HST) and **(B)** control session (CON). MVIC gradually returned to baseline values by 1 h and super-compensation took place as soon as 6 h post-training, which was earlier than the current super-compensation model (Bompa and Haff, 2009). No significant main effects were observed across time for CON (*P* = 0.154). *Indicates a significant main effect over time while ^#^indicates a moderate to large effect size.

Figure 2 shows the change in MVIC for HST and CON across all time points. One-way ANOVA showed a main effect of time for HST (*F*_(10,120)_ = 10.185, *P* < 0.001). *Post hoc* analyses revealed a significantly lower MVIC immediately post training compared to baseline (−19.5%, *p* = 0.001). Moderate and large effects were detected at 10 min post (−13.7%, *d* = 1.30, 95% CI [0.29, 1.91]), 20 min post (−8.1%, *d* = 1.14, 95% CI [0.08, 1.48]) and 30 min post (−5.7%, *d* = 0.71, 95% CI [0.31, 1.22]). A significant increase in force was observed at 24 h (8.5%, *p* = 0.003), and large effect sizes were also detected at 6 h (5.6%, *d* = 0.89, 95% CI [0.36, 1.16]) and 48 h (10.0%, *d* = 1.34, 95% CI [0.38, 1.14]). Changes in maximal force for the control condition are shown in Figure 2B. No main effect was detected for CON (*F*_(10,130)_ = 1.746, *P* = 0.154).

### Peripheral Nerve Excitability

**Figure 3:**
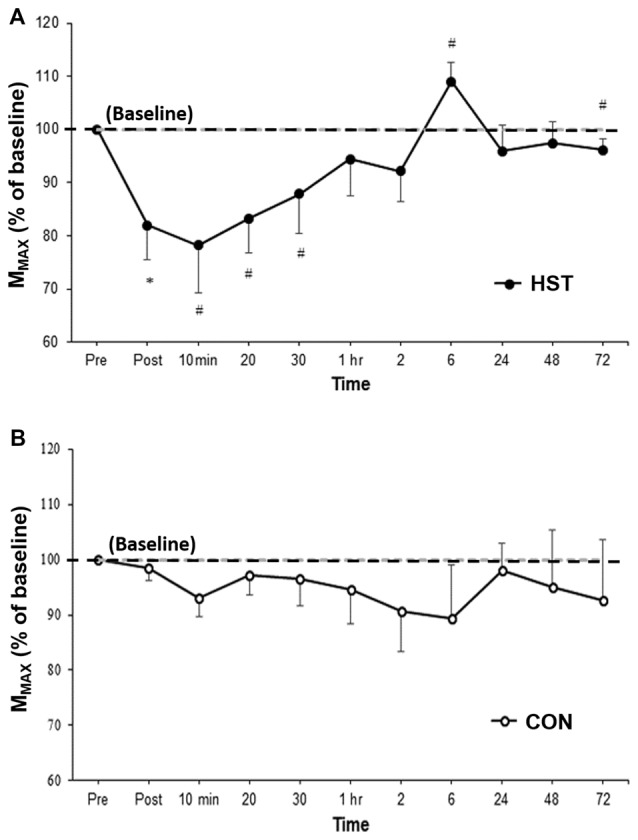
**Changes in M_MAX_ as a percentage of baseline values for (A)** HST and **(B)** CON. Similar to MVIC, M_MAX_ gradually returned to baseline values by 1 h with a super-compensation effect taking place at 6 h post-training. No significant main effects were observed across time for CON (*P* = 0.623). *Indicates a significant main effect for training over time while ^#^indicates a moderate to large effect size.

**Figure 4:**
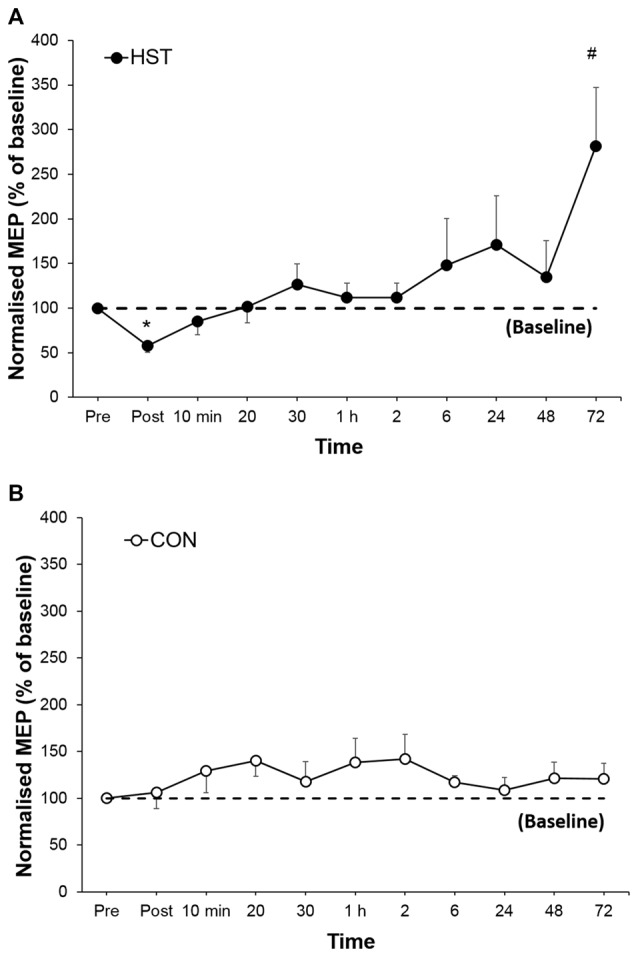
**Changes in normalized single-pulse motor-evoked potential (MEP) for (A)** HST and **(B)** CON. Reductions in the training group MEP immediately post-training indicate initial changes in corticospinal drive followed by an increase in corticospinal drive in the later stages of the super-compensation cycle. *Indicates a significant main effect for training over time while ^#^indicates a moderate to large effect size. No significant main effects were observed across time for CON (*P* = 0.627).

Figure 3 shows the change in M_MAX_ in HST and CON across all time points. One way ANOVA showed a main effect of time for the strength condition (*F*_(2.475, 29.705)_ = 3.179, *P* = 0.047). *Post hoc* analysis revealed M_MAX_ was significantly lower compared to baseline immediately post training (−21.4%, *p* = 0.004). Moderate and large effects were detected at 10 min post (−20.4, *d* = 0.84, 95% CI [0.11, 1.41]), 20 min post (−15.9, *d* = 0.88, 95% CI [0.05, 1.59]) and 30 min post (−13.3%, *d* = 0.66, 95% CI [0.24, 1.27]). M_MAX_ returned to baseline at 1 h with moderate and large effects detected at 6 h (27.9%, *d* = 0.94, 95% CI [0.36, 1.13]) and 72 h (−3.9%, *d* = 0.69, 95% CI [0.51, 0.97]). Changes in M_MAX_ for the control condition are shown in Figure 4B. No main effect was detected for CON (*F*_(10,120)_ = 0.443, *P* = 0.623).

### Corticospinal Excitability

**Figure 5:**
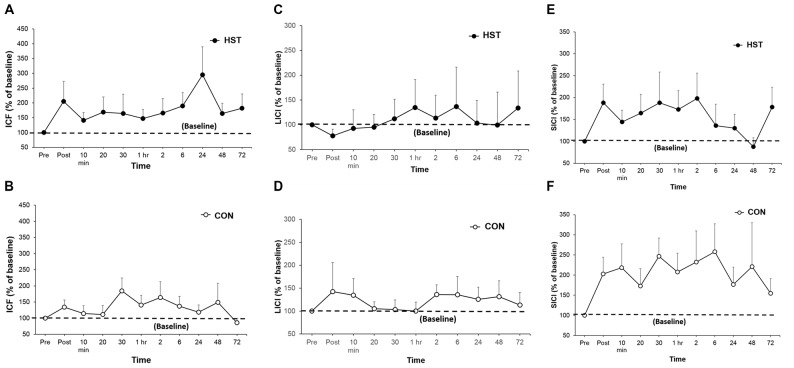
**A comparison of post-training transcranial magnetic stimulation (TMS) measures for intra-cortical facilitation (ICF), long-interval intra-cortical inhibition (LICI) and short-interval intra-cortical inhibition (SICI) in HST and CON.** No significant main effects were observed for ICF **(A,B)**, LICI **(C,D)** and SICI **(E,F)** were observed in both groups over time.

Figure 4 shows the change in normalized MEP amplitude for HST and CON across all time point. One way ANOVA showed a main effect of time for the strength condition (*F*_(10,110)_ = 3.336, *P* = 0.001). *Post hoc* analysis revealed a large decrease in MEP compared to baseline immediately post training (−46.1%, *p* < 0.001) gradually returning to pre training levels at 1 h and a large increase (181.3%, *d* = 1.04, 95% CI [0.46, 2.12]) at 72 h. Changes in MEP for the control condition are shown in Figure 5B. No main effect was detected for the control condition (*F*_(10,110)_ = 0.739, *P* = 0.661).

### ICF and Inhibition

Figure 5 shows the time-course of ICF, LICI and SICI following HST and CON. One-way ANOVA showed no main effect of time for the ICF (*F*_(10,110)_ = 0.750, *P* = 0.676), LICI (*F*_(10,110)_ = 0.838, *P* = 0.497) and SICI (*F*_(10,110)_ = 0.716, *P* = 0.582) in the HST group. No main effect was detected for the ICF (*F*_(10,110)_ = 1.193, *P* = 0.328), LICI (*F*_(10,110)_ = 0.688, *P* = 0.586) and SICI (*F*_(10,110)_ = 0.482, *P* = 0.709) for CON as well.

## Discussion

The aim of the study was to map the time-course of corticospinal adaptations and maximal force output, up to 72 h post-training, following a single session of HST of the BB. The results indicated an immediate reduction in MVIC, followed by a super-compensatory increase at 6 h post-training. M_MAX_ showed a reduction immediately post-training that lasted for 30 min, before increasing at 6 h. A reduction in MEP amplitude was observed immediately post-training up to 20 min, which was followed by an increase at the 48 and 72 h mark. Collectively, our results suggest that changes in corticospinal excitability, intra-cortical inhibition and facilitation, peripheral nerve excitability and maximal force production following HST of the BB may follow a shorter time-course of fatigue, recover and super-compensation as previously suggested by Bompa and Haff (2009).

The main finding from this study showed that MVIC returned to baseline levels within 1 h, with an increase at 6 h post-training indicative of a super-compensation effect. This pattern of recovery within 1 h and super-compensation by 6 h post-training suggests a significantly shorter time-course of recovery and super-compensation compared to the current proposed model. A possible explanation for this effect may be that training in the morning may improve performance measures later in the day. A study by Cook et al. (2014) found that when a 3 RM back squat protocol was performed in the morning, strength was improved when tested in the afternoon. Similarly, Ekstrand et al. (2013) showed that resistance exercise in the morning improved explosive power 4–6 h later. However, afternoon and evening strength training can be positively influenced by the body’s natural circadian rhythm and optimal core temperature (Teo et al., 2011a,b) and thus may contribute at least in part to these findings. Previously, the super-compensation model has suggested a significant reduction in force output observed immediately post-training, remaining impaired up until 1 h (Ruotsalainen et al., 2014), followed by a proposed return to baseline between 24 h and 48 h (Ide et al., 2011). It is therefore recommended that a 72 h rest period between HST sessions to prevent overtraining. This concept is in line with work by Howatson et al. (2016) suggesting force is impaired until at least 24 h after strength exercise. However, studies prescribing strength training frequencies as high as five times per week have also shown greater increases, maximum bench press than those who trained four or less times per week (McKenzie, 1981; Serra et al., 2015). Other high frequency protocols (Raastad et al., 2012) have also supported the idea of increased training frequency. The current inconsistencies on performance recovery, improvement and training frequency is likely due to the differing protocols used in previous literature thus making comparison between studies difficult. The findings of the current study suggest a shortened time course of recovery with strength training in the arm and supports previous research (McKenzie, 1981; Raastad et al., 2012; Serra et al., 2015) applying high frequency training for optimal strength gains.

Similarly, the observed changes in M_MAX_ post-training do not adhere to the time-course as shown in previous super-compensation models (Bompa and Haff, 2009). When analyzed over time, there was a significant suppression of M_MAX_ post-training lasting up until 30 min. M_MAX_ has previously been shown to decline with muscle fatigue in the elbow flexors with maximal contractions (Todd et al., 2003). A likely reason for the suppression of M_MAX_ may be explained by a reduction in sodium-potassium pump efficiency of the sarcolemma following HST (Kirkendall, 1990; Nielsen and Clausen, 2000; Tucker et al., 2005; Mileva et al., 2012). Other mechanisms have also been implicated, such as fatigue-related changes in neurotransmitter release at the neuromuscular junction that may affect electrical nerve conduction (Kirkendall, 1990; Deschenes et al., 1994). Following this, increases in M_MAX_ showed an excitatory increase above baseline as early as 6 h whereby increased peripheral drive may reflect alterations in motor neuron recruitment and firing rate (Aagaard, 2003). Similar to force production, the time-course of M_MAX_ fatigue, recovery and super-compensation was much shorter than proposed by previous literature (Bompa and Haff, 2009) and coincides with an increase in force generating capacity. The super-compensation window appears to begin earlier than the proposed time line in this model. The efficacy of peripheral nerve excitability recovery may contribute, at least in part, to the shortened time-course for super compensatory effects after a single strength training session.

In this study, normalized MEP responses from TMS showed an immediate decrease in corticospinal excitability that lasted up to 30 min post-training. Previous studies have showed mixed evidence on the changes in MEP amplitude following HST (Kidgell et al., 2010; Weier et al., 2012; Hendy and Kidgell, 2013; Ruotsalainen et al., 2014). Ruotsalainen et al. (2014) reported an increase in MEP amplitude at the start of a task, while McNeil et al. (2011a) showed a decrease in MEP amplitude after sustained muscular effort at 25% of MVC over 10 min. Our study showed a significant reduction in MEP amplitude immediately post-training, followed by an increase at 72 h. Our findings are in agreement with previous studies (Todd et al., 2003; McNeil et al., 2011a) where a reduction in MEP amplitude following sustained submaximal muscular contractions in the elbow flexors was observed (Todd et al., 2003), and has similarly been found in other muscle groups after isometric voluntary contractions (McNeil et al., 2011a).

An interesting finding from our study was the lack of intra-cortical changes after a single session HST. This is dissimilar to studies reporting an increase in corticospinal excitability and a reduction in SICI after 2 and 4 weeks of strength training in the upper and lower limb (Weier et al., 2012; Hendy and Kidgell, 2013). Our findings suggest that, with no evident change in ICF or inhibition and a concurrent decrease in peripheral neural excitability the reductions in MEP amplitude appear to be primarily driven by mechanisms downstream of the M1. It is possible that spinal inhibitory mechanisms contribute to this finding (McNeil et al., 2011a). Further, changes in excitability have commonly been found to occur at sub-cortical spinal levels with acute and early strength training (Aagaard, 2003; Nuzzo et al., 2016). Therefore the acute responses may reflect perturbations at subcortical levels.

In light of our findings that differ to previous studies, we acknowledge that several limitations in our study that may have contributed to disparate findings. First, one strength session may not be a sufficient stimulus to induce long-lasting changes, but rather, changes downstream of the M1 may primarily drive super-compensatory responses after training. Second, maximal force production has been shown to increase with training, without any apparent increases in sEMG (Cannon and Cafarelli, 1987; McNeil et al., 2011a) and that maximal voluntary activation increases may not be reflected in sEMG signals (Latella et al., 2012). This would imply that any increase could not be completely attributed to changes in central nervous system. Third, HST recruits small and large motor units and testing the corticospinal tract at rest with TMS may target different neurons in the motor neuron pool and not be a direct representation of activated motor units (McNeil et al., 2011a). Likewise, the difference between gross movement employing large muscle groups, synergists and stabilizers in comparison to simple or isolated tasks is not known, and it is possible that exercise complexity may influence the output from the central nervous system. Although the primary aim of this study was to investigate acute neurophysiological responses, other factors outside the scope of this study such as mechanical, metabolite and hormonal responses may also contribute to the super-compensation cycle. Fourth, it is believed that alteration of the LICI inter stimulus interval from 100 ms to 150 ms may influence the activation of pre and post synaptic GABA_b_ receptors and thus should be considered to clarify the locus of GABA_b_ mediated inhibition (Vallence et al., 2014). Lastly, we acknowledge that the findings support a shortened super-compensation cycle in only recreationally trained populations and may not translate to other populations such as older adults or even in athletes of different training status. Future studies should compare different population groups; novice, or elite, and within training factors such as the effects of increased volume, which may present a different fatigue and recovery response profile due to physiological factors outside the scope of this study.

In conclusion, our findings reveal that after a single strength training stimulus, the time-course of fatigue and recovery and possible super-compensation from an acute HST session in the BB appears to be shorter than that proposed in the current super-compensation model for recreationally trained populations. We acknowledge that other factors may also contribute to the super-compensation cycle, however, the observed neurophysiological changes appear to be primarily driven by peripheral neural mechanisms downstream of the M1. Our results from this study may have significant implications for coaches and strength and power athletes who may program their training based on the current super-compensation model. Based on our current findings, it may be that optimal frequency of strength training can be scheduled sooner than 72 h to enhance strength and neuromuscular adaptations associated with HST. We believe that investigating the basic of post-training neurophysiological changes and comparing it to the super-compensation model may provide evidence for better exercise prescription in future.

## Author Contributions

CL, AJP and DVW conceptualized the study design. CL, AMH and W-PT collected and analyzed the data. All authors contributed equally to the write-up of this manuscript.

## Conflict of Interest Statement

The authors declare that the research was conducted in the absence of any commercial or financial relationships that could be construed as a potential conflict of interest.
